# Typing of human rotaviruses: Nucleotide mismatches between the VP7 gene and primer are associated with genotyping failure

**DOI:** 10.1186/1743-422X-2-24

**Published:** 2005-03-24

**Authors:** Mustafizur Rahman, Rasheda Sultana, Goutam Podder, Abu SG Faruque, Jelle Matthijnssens, Khalequz Zaman, Robert F Breiman, David A Sack, Marc Van Ranst, Tasnim Azim

**Affiliations:** 1 ICDDR,B: Centre for Health and Population Research, Mohakhali, Dhaka-1212, Bangladesh; 2 Laboratory of Clinical and Epidemiological Virology, Rega Institute for Medical Research, University of Leuven, B-3000, Leuven, Belgium

## Abstract

**Background:**

Rotavirus genotyping is performed by using reverse transcription PCR with type-specific-primers. Because the high rotavirus mutation rate generates an extensive genomic variation, different G-type-specific primer sets are applied in different geographical locations. In Bangladesh, a significant proportion (36.9%) of the rotavirus strains isolated in 2002 could not be G-typed using the routinely used primer set. To investigate the reason why the strains were untypeable, nucleotide sequencing of the VP7 genes was performed.

**Results:**

Four nucleotide substitutions at the G1 primer-binding site of the VP7 gene of Bangladeshi G1 rotaviruses rendered a major proportion of circulating strains untypeable using the routine primer set. Using an alternative primer set, we could identify G1 rotaviruses as the most prevalent genotype (44.8%), followed by G9 (21.7%), G2 (15.0%) and G4 (13.8%).

**Conclusion:**

Because of the natural variation in the rotaviral gene sequences, close monitoring of rotavirus genotyping methods is important.

## Background

Rotaviruses remain the most common cause of acute gastroenteritis worldwide and cause an estimated 600,000 deaths in children less than 5 years of age [20]. The high disease burden motivated major efforts to develop a suitable rotavirus vaccine. However, the vaccine efficacy is being challenged by the extensive strain diversity of the rotaviruses [3,7-9,13,14].

Rotaviruses belong to the *Reoviridae*, and their genome consists of 11 segments of double stranded RNA. The gene segment coding for the VP7 glycoprotein is the basis for genotyping group A rotaviruses into at least 15 G-genotypes. Among them, G1, G2, G3, G4 and G9 are the most common G-types in humans [5,15,16,19,21,23]. The importance of type-specific immunological protection against rotavirus disease is still under discussion [13].

G-genotyping is performed using type-specific-primer-based RT-PCR. Two common primer sets introduced by Gouvea et al. [6] and Das et al. [2] are currently used in rotavirus G-typing surveillance programs [22]. A failure to genotype or mistyping has already been reported from different parts of the world. These reports showed that nucleotide sequence differences between the target region of the respective genes and the primer sequences used for typing led to the genotyping failure [1,10,11,17].

The Dhaka hospital of ICDDR,B, situated in the central Bangladesh, and the Matlab hospital, located 45 km south east of Dhaka respectively treat about 100,000 and 15,000 diarrhoeal patients each year. A hospital surveillance system has been established in these hospitals by ICDDR,B to collect information on clinical, epidemiological and demographic characteristics of the patients attending the hospital since 1978. In Bangladesh, rotavirus strains have previously been typed using a variety of techniques. Serotyping was introduced by Ward et al. [28] with specimens collected during 1985–1986 in Dhaka using neutralization with hyperimmune antisera against prototype rotavirus strains G1, G2, G3 and G4. They concluded that epitopic variations between rotavirus strains influenced the sensitivity of serotyping. Fun et al. [4] detected the major rotavirus types (G1 to G4) by RNA hybridization with serotype-specific synthetic oligonucleotide probes, but this method could not type 33.3% of the Bangladeshi rotaviruses. Likewise, RT-PCR depending on type-specific oligonucleotide primers failed to type a significant portion of rotaviruses. Rotavirus surveillance studies in Bangladesh between 1987 and 1997 reported that 1,095 (43.7%) samples out of 2,515 were G-untypeable [25-27].

In this study, we characterized rotavirus positive stool specimens collected in the Dhaka and Matlab hospitals during 2002 by using RT-PCR based on the primer set described by Das et al. [2]. We found that a major proportion of the specimens were untypeable. Nucleotide sequences of VP7 genes were performed to investigate the reason why they were untypeable with the routine primer set. The untypeable specimens were further characterized by using a different primer set described by Gouvea et al. [6].

## Results and Discussion

### Detection of rotavirus strains

In 2002, a total of 3,803 patients with history of watery diarrhea were included in the hospital surveillance system. In Dhaka and Matlab, 535 (27.2%) and 358 (19.4%) specimens were positive for group A rotavirus antigens by enzyme immunoassay.

### G typing using the Das primer set

Rotavirus G-typing was carried out for all rotavirus positive specimens from Matlab and for every fourth of the rotavirus-positive specimens from Dhaka. Some samples were excluded from this study due to unavailability of sufficient amount of stool specimens for testing. G-typing was performed on 433 rotavirus ELISA-positive stool samples by RT-PCR using the primer set described by Das et al. [4], which was routinely used in our laboratory. The most prevalent G types were G9 (20.5%); G2 (14.6%), and G4 (13.8%). G1 comprised only 11.6% of the isolates and 36.9% of the rotavirus-positive samples were untypeable.

### VP7 gene sequence analysis

**Figure 2:**
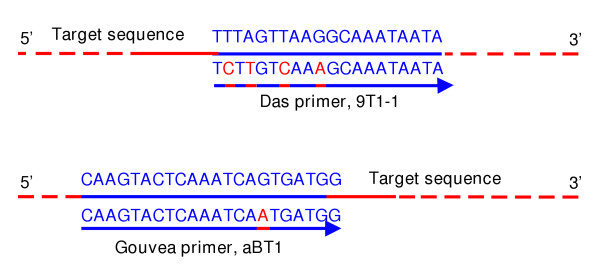
Nucleotide mismatches in the primers. The target sequence is the VP7 gene of G1 Bangladeshi strains. The G1 rotavirus VP7 gene specific primers were described by Das et al. [2] and Gouvea et al. [6]. Mismatches are in red.

We amplified the VP7 genes of five randomly selected untypeable strains (Dhaka162-02, Dhaka18-02, Dhaka164-02 Dhaka165-02 and Matlab26-02) using the VP7 consensus primers Beg9-End9 as described by Gouva et al. [6] and sequenced their complete open reading frame [GenBank:AY631050, GenBank:AY631054]. They were typed as G1 rotaviruses by using BLAST homology searches (99–100% nucleotide and amino acid identities with the Indian G1 rotavirus strain, ISO-4). To compare them with the typeable G1 sequences, the VP7 genes of two typeable G1 strains, Dhaka8-02 [GenBank:AY631049] and Matlab159-02 [GenBank:AY631055] were sequenced. We found that the nucleotide sequences of the typeable and untypeable G1 strains were 100% identical at the G1 primer-binding sites. We aligned the target G1 VP7 sequence with the Das G1 primer sequence (reverse primer, 9T1-1; 5'-TCTTGTCAAAGCAAATAATG-3'; nt 176–195, prototype strain Wa [GenBank:M21843]) to determine if there was any mismatch between them. Four mismatches were found in the Das G1 primer, 9T1-1, at the 5' end (Fig. 2). Due to these mismatches, the Das G1 primer failed to detect most (75%) of the G1 strains. Since, the primer set had perfect matches at the 3' end, it could detect 25% of the G1 rotaviruses. When we compared the target sequence with the Gouvea G1 primer sequence (forward primer, aBT1; 5'-CAAGTACTCAAATCAATGATGG-3'; nt 314–335, prototype strain Wa), we found only one mismatch (Fig. 2). Therefore, the Gouvea G1 primer was found to be more suitable for typing our G1 strains.

### Distribution of G types using Gouvea primer set

**Figure 1:**
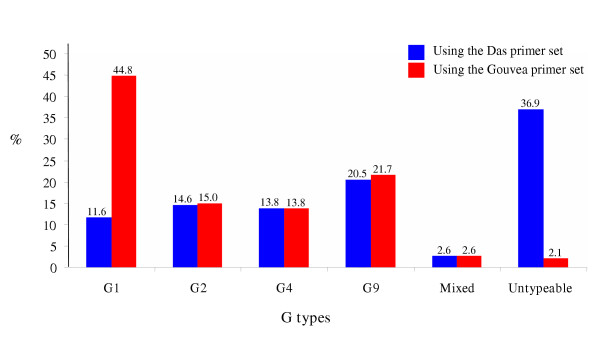
Effect of untypeable strains on G typing of Bangladeshi rotavirus strains isolated in 2002 (n = 433).

The untypeable specimens were typed using the primer set described by Gouvea et al. [6]. After typing with the Gouvea primer set, the distribution of rotavirus G-types changed dramatically (Fig. 1). Type G1 now accounted for 44.8% of the isolates and became the most prevalent genotype, and the number of untypeable strains was reduced from 36.9 to 2.1 %. The other common G types were G9 (21.7%), G2 (15.0%), and G4 (13.8%). The previous studies in Bangladesh reported that G4 strains were the most prevalent strains during 1992–1997 and a significant number of rotavirus strains were untypeable using the Das primer set [26]. It is likely that the Das primer set could not detect most of the G1 rotaviruses in the previous years and that a majority of the untypeable rotaviruses were G1 strains.

## Conclusion

Because of the natural variation in the rotaviral gene sequences, close monitoring of rotavirus genotyping methods is important. The findings described in this paper will be important for genotyping strategies in the rotavirus surveillance studies.

## Materials and methods

### Sample collection

Stool specimens were collected from patients who presented with diarrhea to the Dhaka and Matlab hospitals of ICDDR,B in 2002. In the Dhaka hospital, stool specimens are routinely collected from every 50^th ^patient and in Matlab hospital, every patient with diarrhea submits a stool specimen for testing.

### Rotavirus antigen detection

Rotavirus antigens (group A-specific VP6 proteins) were detected in the stool specimen using a solid phase sandwich type enzyme immunoassay modelled after Dakopatts commercial kit incorporating rabbit hyperimmune antisera produced at ICDDR,B and an anti-human rotavirus-horseradish peroxidase conjugate (Dakopatts, Copenhagen, Denmark) using the same criteria for determination of positivity as those used by the Dakopatts kit [26].

### RNA extraction

The QIAamp Viral RNA mini kit (Qiagen/Westburg, Leusden, The Netherlands) was used according to the manufacturer's instructions for the extraction of rotavirus RNA from the stool samples.

### RT-PCR

A reverse transcriptase-polymerase chain reaction (RT-PCR) was carried out using the Qiagen OneStep RT-PCR Kit (Qiagen/Westburg) as previously described by Das et al. [2] and Gouvea et al. [6] for rotavirus G-types (G1, G2, G3, G4 and G9) using type-specific oligonucleotide primers. The reaction was carried out with an initial reverse transcription step at 45°C for 30 min, followed by 35 cycles of amplification (30 sec at 94°C, 30 sec at 50°C, 1 min at 72°C), and a final extension of 7 min at 72°C in a thermal cycler (Eppendorf, Hamburg, AG). PCR products were run on a 2% agarose gel, and stained with ethidium bromide. Specific segment sizes for the different G types were visualized under UV-light.

### Nucleotide sequencing

The amplified PCR products were purified with the QIA quick PCR purification kit (Qiagen/Westburg), and sequenced in both directions using the dideoxy-nucleotide chain termination method with the ABI PRISM^® ^BigDye Terminator Cycle Sequencing Reaction kit (Perkin-Elmer Applied Biosystems, Foster City, California) on an automated sequencer (ABI PRISM™ 3100). The Beg9 and End9 RT-PCR primers were used as sequencing primers.

### Sequence analysis

The chromatogram sequencing files were inspected using Chromas 2.2 (Technelysium, Queensland, Australia), and consensus sequences were prepared using SeqMan II (DNASTAR, Madison, WI). Multiple sequence alignments were performed using CLUSTALX 1.81 [24]. Sequences were manually edited in the GeneDoc version 2.6.002 alignment editor [18].

### Sequence submission

The nucleotide sequence data were deposited in GenBank using the National Center for Biotechnology Information (NCBI, Bethesda, MD) Sequin 5.15 submission tool  under accession numbers AY631049-AY631055.

## Competing interests

The author(s) declare that they have no competing interests.

## Authors' contributions

MR carried out the laboratory tests and wrote the manuscript; RS and GP carried out RT-PCR tests; JM performed the sequencing experiments; AF, KZ, RB and DS supervised the rotavirus surveillance program and critically revised the manuscript; MVR and TA supervised the study.
